# Cardiac Transplantation and the Use of Cannabis

**DOI:** 10.3390/life11101063

**Published:** 2021-10-09

**Authors:** Hirak Shah, Meg Fraser, Arianne C. Agdamag, Valmiki Maharaj, Bellony Nzemenoh, Cindy M. Martin, Tamas Alexy, Daniel J. Garry

**Affiliations:** 1 Lillehei Heart Institute and Cardiovascular Division, Department of Medicine, University of Minnesota, Minneapolis, MN 55455, USA; hshah6@kumc.edu (H.S.); agdam001@umn.edu (A.C.A.); maha0104@umn.edu (V.M.); cmmartin@umn.edu (C.M.M.); 2 Advanced Heart Failure Program, Mechanical Circulatory Support Service and Cardiac Transplant Program, University of Minnesota, Minneapolis, MN 55455, USA; Mfraser10@umphysicians.umn.edu; 3 Department of Medicine, University of Minnesota, Minneapolis, MN 55455, USA; nzeme001@umn.edu; 4 Paul and Sheila Wellstone Muscular Dystrophy Center, University of Minnesota, Minneapolis, MN 55455, USA

**Keywords:** heart transplantation, cannabis, tetrahydrocannabinol, survey

## Abstract

Cardiac transplantation requires the careful allocation of a limited number of precious organs. Therefore, it is critical to select candidates that will receive the greatest anticipated medical benefit but will also serve as the best stewards of the organ. Individual transplant teams have established prerequisites pertaining to recreational drug, tobacco, alcohol, and controlled substance use in potential organ recipients and post-transplantation. Legalization of cannabis and implementation of its prescription-based use for the management of patients with chronic conditions have been increasing over the past years. Center requirements regarding abstinence from recreational and medical cannabis use vary due to rapidly changing state regulations, as well as the lack of clinical safety data in this population. This is evident by the results of the multicenter survey presented in this paper. Developing uniform guidelines around cannabis use will be imperative not only for providers but also for patients.

## 1. Introduction 

**Figure 1:**
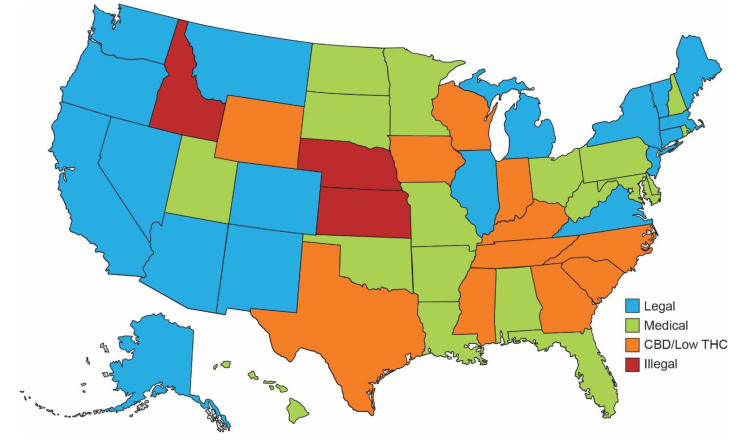
Legality of cannabis by state within the US. The map outlines the current legal status of cannabis based on individual policies of the respective states. The blue color represents states where both recreational and medical cannabis use are legal. States are shown in green where medical cannabis is legal. Orange represents states where recreational and medical cannabis are illegal; however, CBD oil/low THC products are legal. Red color highlights states where any type of cannabis is considered illegal. Map is considered up to date as of 30 August 2021. The figure has been adapted in part from the following reference [2].

Cannabis, also referred to as marijuana, is one of the most widely used substances in the United States [1]. Its medical and recreational use in potential heart transplant recipients remains a highly controversial topic and is subject to ongoing debates. Unfortunately, there are limited guidelines for clinicians to reference when considering patients with active cannabis use for cardiac transplant candidacy. The majority of states within the US have passed laws legalizing cannabis for medicinal and/or recreational use (Figure 1) [2,3]. In addition, multiple states have passed legislation mandating that medical cannabis use cannot be the sole disqualifying factor in determining candidacy for heart transplantation [4]. However, there is no current uniform policy to guide the transplant eligibility of patients who are active cannabis users. The International Society for Heart and Lung Transplantation states that “each center will need to develop its own specific criteria for adjudicating candidacy for marijuana users” [5]. Given the changes in legislation involving cannabis and its rapidly increasing use, it is imperative that the heart failure/cardiac transplant community form a uniform consensus on the criteria that define the transplant eligibility of these patients. The purpose of this review is to summarize the literature on the cardiovascular effects of cannabis in transplant recipients and to highlight the challenges patients and transplant programs face.

## 2. Physiology of the Endocannabinoid System (ECS)

**Figure 2:**
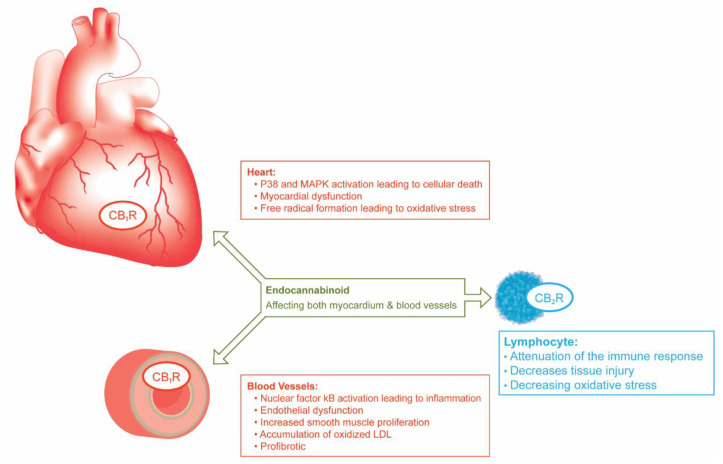
Various effects of cannabinoids on the cardiovascular system. The schematic also highlights that myocardial and endothelial cells express cannabinoid receptor 1 (CBR1), while lymphocytes express cannabinoid receptor 2 (CB2R). The figure has been adapted in part from the following reference [19].

Cannabis is derived from the *Cannabaceae* plant species and is widely known as cannabis [6]. Although its two major active ingredients are tetrahydrocannabinol (THC) and cannabidiol (CBD), the substance itself is complex and is comprised of more than 480 individual compounds [7]. THC and CBD exert their effects primarily via the following two cellular receptors: cannabinoid receptor 1 (CB1R) and cannabinoid receptor 2 (CB2R) [8]. CB1R is the most abundant G-protein-coupled receptor in the mammalian brain with a widespread expression profile, including the basal ganglia, substantia nigra pars reticulata, cerebellum, hippocampus, and cerebral cortex [9]. Therefore, it is the receptor that primarily promotes the psychotropic effects of cannabis. However, CB1R is also expressed in peripheral tissues, including the myocardium, vascular endothelial/smooth muscle cells, and vagal afferents [10]. In contrast, CB2R is found primarily in immune cells, osteoblasts, and osteoclasts [11,12]. Activation of this receptor does not have known direct downstream cardiovascular effects, but rather has an immunomodulatory role (Figure 2) [13]. THC serves as an agonist of both CB1R and CB2R and is, therefore, the primary ingredient responsible for the psychotropic effects of cannabis. CBD has no psychoactive properties as it does not stimulate CB1R but was shown to exert anti-inflammatory and antioxidant properties in various models and tissues [14,15,16,17,18].

## 3. Activation of CB1R (THC) May Provoke Detrimental Cardiovascular Effects While CB2R Activation May Be Cardioprotective

The majority of adverse cardiovascular effects of cannabis are mediated by THC via CB1R activation [19]. Downstream signaling may promote reduced cardiac contractility, endothelial dysfunction, vascular smooth muscle proliferation, increased fibrosis, accelerated atherosclerosis, and decreased insulin sensitivity [19,20]. CB1R agonists were shown to upregulate angiotensin-1 receptor (AT1R) expression and to promote reactive oxygen species generation in endothelial and vascular smooth muscle cells [21,22]. Both processes are associated with accelerated atherosclerosis. Furthermore, unstable arterial plaques are known to have higher CB1R expression [19]. Not surprisingly, CB1R inhibition leads to decreased vascular AT1R expression, improves endothelial function, slows the progression of atherosclerosis, attenuates cellular death, and reduces inflammation and adverse tissue remodeling [21,23,24]

CB2 receptors are expressed predominantly outside of the central nervous system on peripheral immune cells, especially B lymphocytes [25]. Agonists of CB2R were shown to attenuate the inflammatory response by reducing cytokine production and oxidative stress and are considered cardioprotective [19,26,27]. Specifically, CB2R up-regulation has been detected in endothelial and smooth muscle cells stimulated by pro-inflammatory triggers, and in myocardium of patients with chronic heart failure [19]. In addition, CB2R activation may reduce atherosclerotic plaque formation, infarct size, ventricular arrhythmias, and collagen deposition following ischemia-reperfusion injury [28,29,30,31].

## 4. Route of Administration and Pharmacokinetics of Cannabis

The cardiovascular effects of cannabis are dependent on several factors, including the composition of the plant (THC content) and the route of administration [19]. The three most common ways to use cannabis are smoking, vaporizing, and oral intake [32]. Smoking cannabis is the most popular method of utilization. THC can be detected in circulation within one minute and may remain detectable in the blood for up to four hours [33,34,35]. In addition, there is an exposure to carcinogens, tar, and carbon monoxide by burning the plant. These increase the risk for bronchitis, pulmonary infections including tuberculosis, and may prompt decreased respiratory function over time [32]. Given the harmful byproducts stemming from smoking cannabis, vaporizing was thought to be a safer alternative. Vaporized cannabis has a similar rate of absorption, peak serum concentration, and half-life as smoked cannabis [36]. There has been a recent increase in cannabinoid-based oil use for vaporization [37]. This practice has led to a marked increase in the incidence of acute lung injury as these products also contain propylene glycol, vitamin E acetate, and other substances implicated in the development of severe pneumonitis [37,38]. In a New England Journal of Medicine publication assessing causes of vaping-associated lung injury, 47 out of 50 patients who developed lung injury from vaping had either detectable THC in their bronchoalveolar lavage fluid or self-reported vaping the substance within 90 days [38]. In fact, the Center for Disease Control and Prevention (CDC) has recommended against vaping THC-containing products due to the associated risk for lung injury. Edible cannabis has a more delayed effect onset (30–60 min) and peak serum concentration is only reached 3 h after ingestion [35]. One relative advantage of edible cannabis is the lack of pulmonary side effects associated with smoking and vaping. However, given the slower onset of action, accurate “dose titration” to achieve the desired effect is more challenging, thereby increasing the risk of overdosing [39]. In addition, different edible forms may have significantly different THC concentrations that render dose titration even more unpredictable. Other pharmaceutical preparations of Cannabis sativa extracts, formulated as oromucosal spray or oily solution packed in soft gelatin capsules, are available in Europe and other countries. These are beneficial in reducing central neuropathic pain in multiple sclerosis and were shown to have an analgesic as well as an anti-inflammatory effect in rheumatoid arthritis [40,41,42]. In addition, there is growing interest in using these products as anticonvulsants for refractory epilepsy, especially in the pediatric population [43].

## 5. Medical Uses of Tetrahydrocannabinol (THC) and Cannabidiol (CBD)

In addition to traditional cannabis, synthetic products containing THC and CBD were also developed. Dronabinol (Marinol) is a THC derivative that is commonly prescribed as an appetite stimulant. It has been used most widely in patients with various malignancies and Human Immunodeficiency Virus (HIV) infection as cachexia is common in these populations. Based on multiple studies, dronabinol has a good reported safety profile and is effective in improving appetite [44]. In patients with advanced heart failure awaiting transplantation, cardiac cachexia is common and is associated with frailty. Some of these candidates are prescribed dronabinol to improve nutritional status; however, post-transplant outcome data are scarce in these patients. Another product that is gaining popularity is CBD or hemp oil. In contrast to THC, CBD/hemp has no psychoactive properties. Based on pre-clinical studies, CBD oil has the potential to benefit patients with chronic pain syndromes and to reduce anxiety and depression [45]. The possible advantages of CBD oil are its very low misuse rate, low addiction potential, and favorable adverse event profile. However, unlike prescription medications, CBD oil has unclear regulations, and, therefore, products obtained from various sources may differ significantly in composition. Given the increasingly widespread use of these products, transplant programs must decide whether to group CBD oil together with cannabis when it comes to prerequisites for heart transplant listing.

## 6. Cardiovascular Effects of Cannabis

Parallel with its increasingly widespread use, the incidence of cannabis-related adverse events is on the rise. Given that cannabis has been designated as a Schedule one drug [46], it is not possible to perform randomized control trials to assess its potential cardiovascular effects within the United States. Consequently, published data stem from case studies or observational studies that are further limited by the facts that cannabis use was self-reported, and the consumed amount could not be accurately quantified [47,48]. In addition, further confounding factors may have been present that are independently associated with adverse clinical outcomes, such as disparities in access to health care, discrepancies in cannabis content, route of administration, duration of use, and co-ingestion with other substances such as opiates, tobacco, alcohol, or other illicit agents [49].

Despite these significant limitations, many studies have demonstrated an association between cannabis use and adverse cardiovascular outcomes as detailed below. It has been shown to increase heart rate and raise blood pressure due to sympathetic nervous system activation [50]. In the acute setting (within one hour of use), the risk of myocardial infarction increased by 4.8-fold [51]. In a large meta-analysis, cannabis use was deemed to be one of the top three triggers to cause myocardial infarction [52]. It was associated with increased cardiovascular mortality in patients 50 years or younger [53] as well as the development of cerebrovascular [54] and peripheral arterial disease [55,56]. In addition, cannabis use may increase the risk for a wide range of cardiac arrhythmias and conduction system abnormalities, such as atrial fibrillation [57], ventricular tachycardia [58,59], and atrio-ventricular block [60].

On the contrary, a few studies have also been published that found no significant association between cannabis use and the incidence of cardiovascular disorders. For example, the CARDIA study enrolled 3617 patients and the use of cannabis was not associated with increased cardiovascular risk after multi-variable adjustments [61]. In summary, there is a potential that using cannabis increases the risk for the development of cardiovascular diseases; however, current studies are observational with significant limitations. Further investigations are necessary to clarify any possible association.

## 7. Cannabis Use and Outcomes in Organ Transplantation

A small number of retrospective studies have assessed transplant outcomes in patients with active cannabis use. These found no detrimental effects on survival or graft function in renal transplant recipients [62]. Cannabis use was also not associated with increased mortality in an unadjusted analysis completed in liver transplant patients [63]. No difference was found in survival, intensive care unit (ICU) utilization, and hospital length of stay after lung transplantation between recipients using and those abstaining from cannabis products [64]. Focusing on heart transplantation, Xu and colleagues found that high risk donor behavior, including cannabis use, had no significant impact on 1- and 5-year survival rates [65]. In summary, more data are needed to accurately evaluate the association between cannabis use and post-transplant outcomes. However, based on small retrospective studies, there is no obvious signal for harm in transplant recipients.

Some of the concerns related to cannabis use in the post-transplant setting include its potential interaction with immunosuppressive agents, increased risk for infections, and cognitive/psychotropic effects. There is evidence that cannabinoids, including CBD oil, may interfere with immunosuppressive therapy, including calcineurin inhibitors [66,67]. Tacrolimus is one of the most widely utilized agents in this setting but has a narrow therapeutic window. Elevated levels are associated with hypertension, renal dysfunction, and neurotoxicity [66]. Cannabis may interact with the activity of the Cytochrome P4503A (CYP3A) enzyme and P-Glycoprotein transporters, potentially leading to severely elevated serum tacrolimus levels [67]. With regard to infections, a number of case reports have documented an association between cannabis use and aspergillosis in transplant recipients [68,69]. Given the psychotropic properties of cannabis and its effect on cognitive function [70], there has been concern that regular consumption may lead to non-adherence with the complicated but essential post-transplant medical regimen. However, this assumption is yet to be comprehensively evaluated (Table 1).

## 8. Transplant Listing and Other Illicit Substance Use

Given the increasingly widespread use of cannabis in the setting of changing regulations, transplant programs will have to establish guidelines regarding its use despite the lack of high-quality data. One approach to cannabis may be to consider it an agent similar to opiates, given that both may be prescribed by medical providers and that they are also used recreationally. In most institutions, the recreational use of opiates is a contraindication to heart transplantation. Centers typically require a period of abstinence (approximately 3 to 6 months) before activating the patient on the wait list and mandate extensive psychosocial evaluation. Testing is performed by a multi-disciplinary team that includes psychiatrists, psychologists, social workers, and transplant coordinators. Although there is no universal testing tool that is utilized throughout the country, some commonly used assessment modalities include the psychosocial assessment for transplantation (SIPAT), psychosocial assessment of candidates for transplantation (PACT), and transplant evaluation rating scale (TERS). In addition, comprehensive urine/blood screening for illicit substances is required on a regular but random basis by most transplant centers. At our institution, patients may be classified into one of the following three categories based on the duration and amount of illicit agent used: (1) substance misuse (3 months of abstinence required), (2) substance use disorder (6 months abstinence is mandated), and (3) recalcitrant substance use disorder that requires 12 months of proven abstinence with a completed rehabilitation program.

## 9. Similarities between Prescription Opiates and Prescription (Medical) Cannabis

There is an ongoing debate within the transplant community whether patients actively taking prescription opiates are appropriate for transplant listing. The majority of transplant centers across the US will proceed and list such patients. The ethical question remains, however, if there is a significant difference between prescribed cannabis and opiate use. Opiates have an addiction potential and have been linked to reduced cognition, decreased medication adherence, and increased risk of death [71,72]. Importantly, a large multi-center study reported that prescription opiates were associated with an increased mortality risk following cardiac transplantation [73]. In contrast, cannabis has a lower potential risk for overdose, less addiction potential, and fewer associated deaths [74,75]. Given that the majority of the transplant community considers prescription opiate use acceptable prior to transplant listing, one may argue cannabis and its derivatives (such as dronabinol) should be treated similarly, especially considering its relatively safer side effect profile and lower addiction potential.

## 10. Current Views on Cannabis Use and Transplantation

In a survey published in 2016, providers from cardiac transplant centers around the world were queried about their policies regarding cannabis use [4]. A total of 360 heart failure providers from 26 countries provided their viewpoint. As expected, there was no uniform consensus on the acceptability of cannabis use in transplant candidates, primarily owing to the lack of high-quality data. Of the participants, 64% stated that patients who use medical cannabis may be listed for heart transplantation. However, only 28% agreed that patients who use recreational cannabis should be listed [4]. It is interesting to see such a stark difference in the perception of medical and recreational cannabis use. This may be driven by the negative stigma associated with the latter. Another concern is the notion that patients using cannabis may have decreased adherence to immunosuppressive therapy, which is known to increase graft rejection rates [76]. However, there are limited data to prove that cannabis use leads to suboptimal medication adherence. In fact, substance use disorder related to alcohol and other illicit substances has not been linked to decreased medication adherence in a population of predominantly liver transplant recipients [77].

## 11. Multicenter Survey on Cannabis Use and Cardiac Transplantation Practices

**Figure 3:**
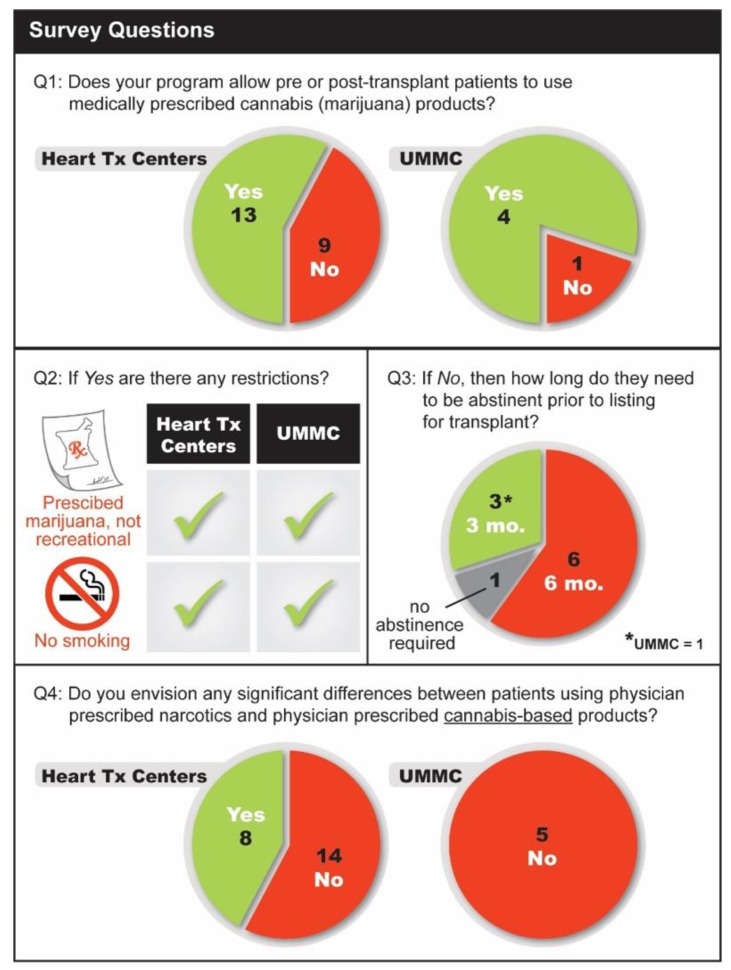
Survey results of 22 US-based heart transplant programs regarding cannabis use in transplant candidates. In addition to these centers, we surveyed the renal, liver, lung, pancreas, intestine, and islet cell transplant programs at the University of Minnesota (UMMC). The survey questions were as follows: (1) Does your program allow pre- or post-cardiac transplant patients to use medically prescribed cannabis (marijuana) products? (2) If yes, are there any restrictions? (3) If no, then how long do they need to be abstinent prior to listing for transplant? (4) Do you envision any significant differences between patients using physician-prescribed narcotics and physician-prescribed cannabis-based products?

Given the vastly different views, we surveyed cardiac transplant centers across the US to assess their approach to recipient cannabis use when considering listing for heart transplantation (Figure 3).

### 11.1. Methods

Twenty-two of the thirty centers approached responded to the email survey request. The programs performed 16 to 132 cardiac transplantations between 2018 and 2019 and were based in California, Texas, Washington, Missouri, North Carolina, Indiana, Tennessee, Georgia, Ohio, Illinois, New York, and Colorado. We posed the following four questions in the email query: (1) Does your program allow pre- or post-cardiac transplant patients to use medically prescribed cannabis (marijuana) products? (2) If yes, are there any restrictions? (3) If no, then how long do they need to be abstinent prior to listing for transplant? (4) Do you envision any significant differences between patients using physician-prescribed narcotics and physician-prescribed cannabis-based products? Given that the questions were sent via email, the investigators were not blinded but the identity of the responders and the responses were kept confidential.

### 11.2. Results

Consistent with a survey published by Neyer et al., there was no consensus regarding cannabis use and listing for heart transplantation [4]. Thirteen programs (59%) would allow patients to use medically prescribed cannabis pre-transplant; however, the majority of them would only permit edible forms as opposed to smoking/vaping it. Nine programs (41%) would not list patients who use any forms of cannabis products pre-transplantation. Six of these programs (67%) require at least six months, two programs (22%) mandate at least three months, and one program (1%) would not require any period of abstinence prior to listing. However, for the program that does not require abstinence, there has to be evidence of decreasing serum THC levels prior to activation on the waitlist. Interestingly, 58% of programs do not perceive a significant difference between prescribed opiates and prescribed cannabis. Among programs that believe that there is a difference, the most common reasons cited were a lack of prescriber oversight when compared to opiates, more limited pharmacodynamics data, and the possible interaction between cannabis and calcineurin inhibitors.

### 11.3. Survey of Other Organ Transplant Programs

We also surveyed the renal, liver, lung, intestine, and pancreas transplant programs at our own institution (UMMC). Four programs (80%) would allow transplant candidates to use medically prescribed cannabis, while one requires three months of abstinence. None of the five programs believed that there is a significant difference between prescribed opiates and medically prescribed cannabis (Question #4).

## 12. Possible Approach to Cannabis Use in Heart Transplant Patients

The care of patients with end stage heart failure is extremely complex and often requires extraordinary measures, such as heart transplantation. Substance use, including cannabis, only adds to this complexity. However, cannabis use is increasing and is legalized in many US states and, therefore, the transplant community must take the initiative to develop guidelines on how to approach these patients. Unfortunately, there is a lack of high-quality data regarding the safety and cardiovascular outcomes associated with cannabis use to guide our decision-making process. Limited observational studies and case reports suggest that cannabis may increase the risk of arrhythmias, myocardial infarction, peripheral vascular disease, aspergillosis, and tacrolimus toxicity. However, these studies are, at most, hypothesis generating as opposed to establishing clear causation. In contrast, observational studies have also been published that demonstrate no increased harm associated with cannabis ingestion. Therefore, at this point, we believe that the approach to recreational cannabis should mirror the view of the transplant community on other substances, such as opiates. We should hold ourselves to the same standards when considering recreational cannabis or other substance use. It would be incongruent for the community to allow the listing of patients who are actively using recreational cannabis but deny listing recreational opiate users. Therefore, patients should demonstrate abstinence from recreational cannabis (3 to 12 months based on their level of use) before activation on the heart transplant waitlist. Additionally, Olt et al. suggested this should be more specific and include the route of administration, frequency of usage, and dependency for future transplant guidelines [78]. As transplant professionals, we bear the duty of enabling our patients to gain access to donor grafts. The delay set forth by the transplant center for listing candidates with cannabis use could indeed have fatal consequences in some patients with end stage heart failure. It is, therefore, critical that patients gain a clear understanding of transplant prerequisites in a timely manner, and it is our duty to provide this education and continued reinforcement. All resources should be provided to candidates, including referral to rehabilitation programs if appropriate. However, it is crucial to emphasize the importance of consistency around minimum requirements within a transplant program.

With respect to prescription/medical cannabis use, policies become even more complex. One potential way to approach the issue is to parallel prescription opiates with prescription cannabis. As described above, opiates have been associated with negative post-transplant outcomes, but their use do not serve as a sole disqualifier for heart transplantation by many centers. Instead, affected patients undergo extensive testing to ensure that they are not abusing the medication, and they can be listed only when the transplant team is assured. Mirroring the approach when considering cannabis use would allow for consistency within the guidelines. If a potential transplant candidate has a valid prescription for cannabis, the team should contact the prescribing provider and discuss whether it is truly necessary to manage the patient’s condition. If it is determined that switching to an alternative medication is not feasible, then the patient should undergo psychosocial testing to exclude substance use disorder. In addition, centers may opt to permit edible forms only, given the known pulmonary side effects of smoking/vaporizing cannabis. If they pass the screening tests, the patient may be activated on the waitlist. The authors acknowledge, however, that it may be difficult to enforce the strict requirement for oral cannabis use, an issue similar to nicotine-containing products. Some centers may opt to also restrict prescription cannabis use as a consequence.

## 13. Conclusions

While the regulations related to cannabis use are constantly evolving, developing a uniform guideline would be imperative not only for providers but also for potential heart transplant candidates. Although it is legal in many states across the US, the transplant community has much to learn about the risks and adverse effects associated with its use. Until more information is available, cannabis should be treated similarly to any other agents with a potential for substance use disorder, such as opiates, alcohol, or tobacco.

## Figures and Tables

**Table 1:** Potential medical concerns related to active cannabis use in transplant candidates.

System	Potential Adverse Effects	References
Cardiovascular	Increased Atherosclerosis and Incidence of Myocardial Infarction	[40,41]
	Arrhythmia (Atrial Fibrillation, Ventricular Tachycardia, AV Block)	[46,47,48,49]
	Cerebral Vascular Accidents	[43]
	Peripheral Arterial Disease	[44,45]
Pulmonary	Decreased Respiratory Function Increased Bronchitis Pneumonitis	[25]
Drug Interaction	Tacrolimus Increased risk of CNI Toxicity	[55,56]
Infectious Disease	Increased risk for Infection (Aspergillosis Pneumonia)	[57,58]
Psychosocial	Decreased Compliance Increased Psychosis	[59]

## Data Availability

Data is contained within the article.
